# Supplementary material for: Mucormycosis: A 14-Year Retrospective Study from a Tertiary Care Center in Lebanon
Source: J Fungi (Basel). 2023 Aug 3;9(8):824. doi: 10.3390/jof9080824 (PMC10456049; doi:10.3390/jof9080824)
Supplement: Supplementary file 1 [file jof-09-00824-s001.zip › jof-2515817-supplementary.pdf]

Variable	Number (%)
Male	25 (58.1%)
Age*	53 (38.0-64.0)
Greater Beirut	17 (39.5%)
Charlson Comorbidity Index*	3 (2.0-5.0)
Hematologic malignancy	29 (67.4%)
AML	14 (32.6%)
ALL	8 (18.6%)
CML	1 (2.3%)
CLL	1 (2.3%)
MDS	6 (14%)
HL	2 (4.7%)
NHL	2 (4.7%)
MM	0 (0%)
Other hematologic malignancy	2 (4.7%)
Diabetes mellitus	15 (34.9%)
Renal failure	5 (11.6%)
Immunocompromise	38 (88.4%)
Solid organ malignancy	3/43 (7%)
Transplant	
SOT	1 (2.3%)
autoBMT	0 (0%)
alloBMT	9 (20.9%)
GVHD	5 (11.6%)
Neutropenia at diagnosis	21 (48.8%)
HIV	0 (0%)
Recent corticosteroids	22 (51.2%)
Recent chemotherapy	30 (69.8%)
Recent immunotherapy	2 (4.7%)
Auto-immune disease	5 (11.6%)
COVID-19	5 (11.6%)
COVID-19 treatment	
Corticosteroids	3 (7%)
Convalescent	1 (2.3%)
Remdesivir	3 (7%)
Baricitinib	0 (0%)
Sotrovimab	0 (0%)
Tocilizumab	1 (2.3%)
Recent antifungals	19 (44.2%)
Fluconazole	6 (14.0%)
Voriconazole	16 (37.2%)
Posaconazole	2 (4.7%)
Itraconazole	0 (0%)
Anidulafungin	1 (2.3%)
Micafungin	0 (0%)
Abelcet	0 (0%)
Ambisome	2 (4.7%)
qSOFA at diagnosis	0 (0.0-1.0)
Presentation of mucormycosis	
Isolated ROCM	27 (62.8%)

Isolated pulmonary	3 (7.0%)
ROCM and pulmonary	3 (7.0%)
ROCM and SSTI	2 (4.7%)
GI	2 (4.7%)
SSTI	6 (14.0%)
Symptoms and signs	
Fever	24/43 (55.8%)
Necrotic eschar	17/43 (39.5%)
Ophthalmoplegia	10/43 (23.3%)
Proptosis	7/43 (16.3%)
Sinusitis	24/43 (55.8%)
Cough	6/43 (14.0%)
Pleuritis	0/43 (0%)
Biopsy	39 (90.7%)
Hyphae on biopsy	32/39 (82.1%)
Site of biopsy	
Palate	5/39 (12.8%)
Sino-nasal	22/39 (56.4%)
Soft tissue	8/39 (20.5%)
Lung	1/39 (2.6%)
Bowel	1/39 (2.6%)
Orbital	2/39 (5.1%)
Culture	10/43 (23.3%)
Source of culture	
Sino-nasal	6/10 (60%)
Soft tissue	1/10 (10%)
Lung	3/10 (33%)
Species	
Not identified	17/43 (39.5%)
Rhizopus	6/43 (14.0%)
Mucor	19/43 (44.2%)
Absidia	1/43 (2.3%)
Imaging	
CT scan	28 (65.1%)
MRI	17/43 (32.6%)
ROCM	32/43 (74.4%)
Imaging findings for ROCM	
Severe soft tissue edema	13/32
Sinus mucoperiosteal thickening	13/32
Bone erosion	8/32
Orbital invasion	14/32
Fat pad thickening/stranding	7/32
Air/fluid level	8/32
Imaging findings of pulmonary mucormycosis	
Consolidation/mass	4/6
Pleural effusion	1/6
Nodules	3/6
Reverse halo	0/6
Air crescent	1/6
Infarcts	2/6

Infiltrates	3/6
Surgical debridement	31 (72.1%)
Days from diagnosis to surgery*	1.0 (0.0-2.0)
Amphotericin B	
Did not receive Amphotericin B	2 (4.7%)
Abelcet	8 (18.6%)
Ambisome	33 (76.7%)
Amphotericin B dose	
5 mg/kg	18 (41.9%)
7.5 mg/kg	17 (39.5%)
10 mg/kg	8 (18.6%)
Antifungal step-down	
Posaconazole	21 (48.8%)
Isavuconazole	1 (11.6%)
Caspofungin	5 (11.6%)
Anidulafungin	2 (4.7%)
No step-down	14 (32.6%)
Duration of Amphotericin B (days)	21 (8.50-36.75)
Adverse events	
Nephrotoxicity	6 (14.0%)
Hepatotoxicity	1 (2.3%)
Electrolytes anomalies	10 (23.3%)
Response to treatment	
Complete response	6 (14.0%)
Stable	17 (39.5%)
Deterioration	20 (46.5%)
In-hospital mortality	20/43 (46.5%)
Time from diagnosis to death	20.50 (7.25-46.5)
Time from diagnosis to discharge in survivors	29.00 (24.0-42.0)

Supplementary table 1. Characteristics of the overall population of patients with mucormycosis. AML, acute myelogenic leukemia; ALL, acute lymphocytic leukemia; CML, chronic myelogenous leukemia; CLL, chronic lymphocytic leukemia; MDS, myelodysplasia; HL, Hodgkin's lymphoma; NHL, non-Hodgkin's lymphoma; MM, multiple myeloma; SOT, solid organ transplant; BMT, bone marrow transplant; GVHD, graft-versus-host disease; ROCM, rhino-orbito-cerebral mucormycosis; SSTI, skin and soft tissue infection; GI, gastrointestinal; CT, computed tomography; MRI, magnetic resonance imaging. *median (interquartile range)
